# Association Between Body Mass Index and Depressive Symptom Trajectories Among Chinese Middle and Old Aged Adults: A Cohort Analysis Based on the CHARLS

**DOI:** 10.1002/brb3.71744

**Published:** 2026-09-15

**Authors:** Dehua Zhao, Xiaoqing Long, Hongying Fan

**Affiliations:** ^1^ Department of Clinical Pharmacy The Third Hospital of Mianyang (Sichuan Mental Health Center) Mianyang China

**Keywords:** BMI, CHARLS, depressive symptoms, GBTM, trajectories

## Abstract

**Background:**

The prevalence of depressive symptoms among middle‐aged and older adults persists at concerning levels. Despite this high prevalence, limited research has investigated the association between body mass index (BMI) and longitudinal trajectories of depressive symptoms. We aimed to examine this understudied association in a nationally representative population‐based longitudinal study.

**Methods:**

The data were obtained from the China Health and Retirement Longitudinal Study (CHARLS), covering the period from 2011 (Wave 1) to 2020 (Wave 5). Depressive symptoms were assessed using the 10‐item Center for Epidemiologic Studies Depression Scale (CESD‐10). We employed group‐based trajectory modeling (GBTM) to analyze depressive symptom trajectories over time, followed by multivariable logistic regressions to examine the association between BMI and depressive symptom trajectories. Gender‐stratified analysis was performed to elucidate differential associations between BMI and trajectories of depressive symptoms across sexes. Additionally, sensitivity analyses were performed to evaluate the stability of the results.

**Results:**

A total of 10,521 participants were included in the final analysis. Two depressive symptom trajectory groups were identified: a stable low‐symptom trajectory group (*N* = 5467, 51.96%) and a consistently high‐symptom trajectory group (*N* = 5054, 48.04%). Compared with normal‐weight, participants with underweight were more likely to suffer from depressive symptoms (OR = 1.29, 95% CI: 1.07–1.56, *p* = 0.009) after adjusting for potential confounding covariates. However, no statistically significant associations were observed in participants with overweight (OR = 0.94, 95% CI: 0.85–1.04, *p* = 0.248) or obesity (OR = 0.89, 95% CI: 0.77–1.02, *p* = 0.100). Gender‐stratified analysis revealed a significant positive association between underweight status and consistently high‐symptom trajectory in males (adjusted OR = 1.32, 95% CI: 1.01–1.72, *p* = 0.041), whereas no statistically significant association was observed in females (adjusted OR = 1.23, 95% CI: 0.94–1.62, *p* = 0.134).

The sensitivity analyses yielded results consistent with our primary findings, thereby reinforcing the robustness of our outcomes.

**Conclusion:**

Underweight was more likely to be associated with depressive symptoms in males than in females. Future investigations are needed to elucidate the underlying biological mechanisms connecting underweight and depressive symptoms, as well as evaluate the potential role of regulating underweight in the prevention and treatment of depressive symptoms.

## Graphical abstract

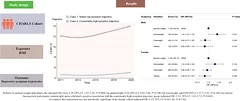

## 1 Introduction

Depressive symptoms, as one of the most common psychiatric disorders among middle‐aged and elderly in worldwide, have prevalence rates of 22.1% in the United States, 34.8% in Japan, 34.6% in France, and 42.0% in China (Chen et al. 2024; Dai et al. 2025; Chang et al. 2025). It is a complex condition characterized by persistent feelings of low mood. In addition to low mood, patients with depressive symptoms often experience various chronic physical diseases, such as cardiovascular diseases, digestive disorders, dementia, metabolic diseases, and cancers (Yang et al. 2025; Zha et al. 2025; Lee and Hong 2025; Liu et al. 2025). Therefore, individuals with depressive symptoms experienced significantly reduced physical functioning and quality of life. According to the previous studies, depressive disorders make up a relatively significant share (4.5%) of the total disability‐adjusted life years and account for 12.1% of the total years lived with a disability globally (Chen et al. 2024). Thus, the overall global burden of depressive symptoms remains severe, and identifying modifiable risk factors is s essential to mitigate the incidence and alleviate the burden of depressive symptoms.

Body mass index (BMI), a widely recognized indicator for assessing nutritional status and health status, has received significant research attention due to its association with depressive symptoms (Luo et al. 2018; Xiao et al. 2025; Zhang et al. 2024). However, results were inconsistent across previous studies. Some found positive associations, others reported negative associations, while still others found no statistically significant or clinically meaningful associations. A population‐based cohort study showed that males with obesity were less likely to suffer from depressive symptoms than males with a normal weight (HR = 0.51, 95% CI = 0.35–0.74), but this result was not observed in females (Luo et al. 2018). Notably, another cohort study demonstrated that BMI was significantly associated with depressive symptoms in females (OR = 0.95, 95% CI: 0.93–0.98) over the long term, whereas no such association was observed in males (Zhang et al. 2024). Moreover, a cross‐sectional study found an S‐shaped association between BMI and depressive symptoms, showing initial decrease followed by significant elevation when exceeding the threshold of 23.22 kg/m^2^ (Xiao et al. 2025). This inconsistency may stem from variations in population characteristics (such as age and race) and confounding factors.

Obesity and depression share common pathophysiological pathways, including chronic low‐grade inflammation, dysregulation of the hypothalamic‐pituitary‐adrenal (HPA) axis, and alterations in adipokine profiles (Dyndał et al. 2025; Soczynska et al. 2011). Beyond these biological pathways, psychosocial mechanisms also play a critical role in the BMI–depression association. Individuals whose body weight deviates from societal norms—whether underweight or overweight—may experience social devaluation, internalized weight bias, and diminished self‐esteem, all of which contribute to depressive symptomatology (Dębski et al. 2024). Furthermore, gender differences may exist in the association between BMI and depressive symptoms. Sex‐specific biological factors, including differences in adipokine profiles, sex hormone regulation, and immune‐inflammatory responses, may contribute to divergent associations between men and women (Ouakinin et al. 2018; Assari and Caldwell 2015). Although previous studies have analyzed the association between BMI and depressive symptoms, they only assessed depressive symptoms at a single time, failing to track the progression of the condition (Luo et al. 2018; Xiao et al. 2025; Zhang et al. 2024). The group‐based trajectory model (GBTM) is a mixture modeling technique that simultaneously estimates multiple developmental trajectories, overcoming the limitations of traditional models that only estimate a single average trajectory for the entire population (Mirza et al. 2016). Therefore, the present study aimed to explore the association of BMI with trajectories of depressive symptoms by using repeated measurements from a national sample of middle‐aged and older Chinese adults. We hope to provide evidence‐based insights to inform policy formulation and intervention strategies for mental health maintenance in middle‐aged and older adults.

## 2 Materials and Methods

### 2.1 Study Population

The data used in this study were obtained from a follow‐up wave of the China Health and Retirement Longitudinal Study (CHARLS), focusing on the middle‐aged and older population in China. Specifically, five waves (2011 wave, 2013 wave, 2015 wave, 2018 wave, and 2020 wave) were utilized for this analysis. The CHARLS is a national longitudinal study conducted by the National School for Development at Peking University, which conducts biennial survey waves to collect data. The first national baseline survey of the CHARLS was conducted between 2011 and 2012. Using multistage probability proportional to size sampling, the study recruited 17,705 respondents across 28 provinces, 150 counties/districts, and 450 villages/resident committees. The respondents were randomly selected and interviewed face‐to‐face in their homes via computer‐assisted personal interviewing technology. Currently, CHARLS data are available only up to the fifth wave (2020), as the sixth wave has not yet been released. Accordingly, the present study used data from waves 1 to 5 of CHARLS. We excluded individuals under age 45, those with missing data on depression assessment or BMI, and those lost to follow‐up. However, individuals who exhibited depressive symptoms or reported a prior diagnosis of emotional, nervous, or psychiatric problems at baseline were not excluded from the analysis. The CHARLS study received ethical approval from the Biomedical Ethics Review Committee of Peking University (IRB00001052‐11015) in accordance with the Declaration of Helsinki. All participants provided written informed consent prior to participation.

### 2.2 Assessment of Depressive Symptoms

Depressive symptoms were assessed using the 10‐item Center for Epidemiological Studies Depression Scale (CESD‐10). The CESD‐10 comprises 10 items, 8 of which assess negative affect, with the remaining 2 items measuring positive affect. Participants were instructed to retrospectively report their mood over the past seven days. To clarify, participants were asked to calculate the specific number of days each symptom occurred, rather than provide an overall frequency assessment. For each symptom, participants were asked to choose one of four response options: (a) rarely or none of the time (<1 day); (b) some or a little of the time (1–2 days); (c) occasionally (3–4 days); or (d) most or all of the time (5–7 days). The total score ranges from 0 to 30, where higher scores indicate more severe depressive symptoms. Previous studies have indicated that the CESD‐10 demonstrates acceptable sensitivity and specificity for identifying depressive symptoms in Chinese populations (Chen et al. 2025; Chen and Mui 2014). Moreover, studies have shown that a cutoff score of ten or higher is indicative of clinically relevant depressive symptoms (Mulugeta et al. 2018; Zhao et al. 2023).

### 2.3 Assessment of BMI

BMI was calculated as body weight (kg) divided by the square of height (m^2^), with both measurements obtained through on‐site assessments at baseline. According to the Chinese criteria for adults (Luo et al. 2018), BMI categories were defined as underweight (<18.5 kg/m^2^), normal‐weight (18.5–23.9 kg/m^2^), overweight (24.0–27.9 kg/m^2^), and obesity (≥ 28.0 kg/m^2^).

### 2.4 Covariates

Drawing from prior research and clinical experience, we strategically selected several covariates to minimize potential confounding effects. The covariates, which included sociodemographic characteristics, health‐related behaviors and factors, and chronic diseases, were obtained from the 2011 baseline survey of CHARLS. Sociodemographic characteristics included age, gender, education level (categorized as elementary school or below, middle school, high school, and college or above), marital status (categorized as married or not married), residence (categorized as rural or urban), health insurance (categorized as urban employee medical insurance, urban and rural resident medical insurance, other medical insurance, or no insurance), and household consumption. Health‐related behaviors comprised drinking status (categorized as drink more than once a month, drink but less than once a month, or do not drink), smoking status (categorized as current smokers, past smokers, or never smokers), social activities, and physical activities (categorized as vigorous, moderate, or other). Health‐related factors included self‐rated health (categorized as good, fair, or poor) and C‐reactive protein (CRP). Chronic diseases included hypertension, dyslipidemia, diabetes, cancer or malignant tumor, chronic lung diseases, liver diseases, heart diseases, stroke, kidney diseases, stomach or other digestive diseases, memory related diseases, arthritis or rheumatism, and asthma.

### 2.5 Statistical Analysis

Continuous variables with normal distributions were presented as mean ± standard deviation (SD), while those exhibiting skewed distributions were summarized using median and interquartile range (IQR). Categorical variables were expressed as frequency counts accompanied by corresponding percentages. Continuous variables were analyzed using either one‐way ANOVA (for normally distributed data) or the Kruskal–Wallis test (for skewed distributions), whereas categorical variables were assessed via chi‐square tests.

Trajectories of depressive symptoms were identified using GBTM, which assumes a censored normal distribution for the repeated CESD‐10 scores to appropriately handle the bounded (0–30) nature of the scale. For each trajectory class, we simultaneously evaluated linear, quadratic, and cubic growth parameters to determine the optimal polynomial order describing symptom progression. Trajectory models with 2 to 6 classes were evaluated to identify the optimal number of depressive symptom trajectories. Model selection was determined through multi‐criteria evaluation: (a) minimization of both Bayesian Information Criterion (BIC) and Akaike Information Criterion (AIC) values, (b) average posterior probabilities (AvePP) ≥0.70 across all trajectory classes, and (c) class membership exceeding 5% of the total sample.

Multivariable logistic regression analyses were conducted to examine association between BMI and depressive symptom trajectories, with results expressed as adjusted odds ratios (ORs) and 95% confidence intervals (CIs). Four hierarchical regression models were constructed through sequential covariate adjustment: Model 1 was a crude model without adjustments; Model 2 was adjusted for sociodemographic characteristics; Model 3 was further adjusted for health‐related behaviors and factors; and Model 4 was additionally adjusted for chronic diseases. Gender‐stratified subgroup analyses were conducted to examine potential gender‐specific association between BMI and depressive symptom trajectories. This approach was motivated by prior studies suggesting that gender may moderate the relationship between BMI and depressive symptoms. To corroborate the robustness of our primary estimates, we conducted three sensitivity analyses. First, we re‐estimated our main analyses after excluding participants with diagnosed memory‐related disorders. Second, to mitigate potential bias stemming from incomplete data, we adopted a complete‐case approach by excluding participants with missing covariate observations. Finally, we calculated *E*‐value to assess the potential influence of unmeasured confounding. Assuming the covariate data were missing at random, we applied multiple imputation by chained equations (MICE) to generate five imputed datasets, thereby reducing selection bias while maintaining statistical power and analytical precision. Statistical analyses employed two‐tailed hypothesis testing with *α* = 0.05 significance threshold. Data analyses were implemented via R software (version 4.3.0).

## 3 Results

### 3.1 Baseline Characteristics of the Study Participants

**FIGURE 1:**
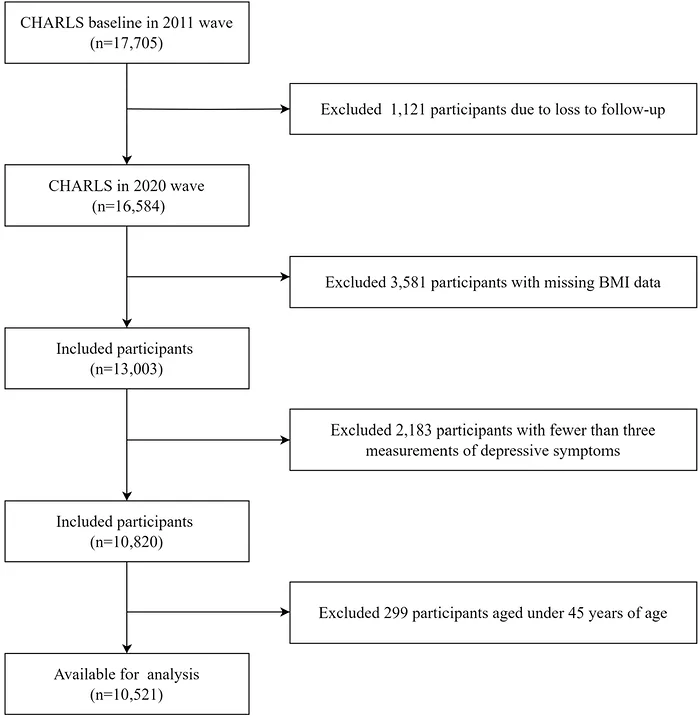
Flow chart of participants selection process.

A total of 17,705 participants were recruited at baseline in 2011 wave. The exclusion criteria comprised several factors. First, 1121 participants were excluded due to loss to follow‐up. Additionally, 3581 participants with missing BMI data were excluded. Furthermore, 2183 participants with fewer than three measurements of depressive symptoms were excluded. Finally, 299 participants aged under 45 years of age were excluded. After applying these exclusion criteria, 10,521 participants with at least three assessment waves of depressive symptoms were retained for final analysis. The participant selection process was shown in Figure 1.

Of the 10,521 participants included in the analysis, the mean age was 57.66 ± 8.41 years old, with 52.14% being female. BMI distribution revealed predominant classification in the normal‐weight range (52.60%, *n* = 5534), followed by overweight (29.80%, *n* = 3135) and obesity categories (11.71%, *n* = 1232). A minimal proportion of participants were classified as underweight (5.89%, *n* = 620). Comparative analyses across BMI groups revealed statistically significant intergroup variations (*p* < 0.05) in all examined covariates, with the exception of liver diseases and memory related diseases. Individuals with higher BMI exhibited a constellation of characteristics. Specifically, they were more likely to be female, of younger age, residing in urban areas, and married; they presented with elevated CRP levels; they demonstrated higher household consumption; their disease history was notable for hypertension, dyslipidemia, diabetes, cancer or malignant tumor, liver diseases, heart diseases, stroke, memory‐related diseases, arthritis or rheumatism, and asthma; and they reported fewer physical activities. The baseline characteristics of the included participants were presented in Table 1.

**TABLE 1:** Baseline characteristics of the included participants.

Variables	Total (*n* = 10,521)	Underweight (*n* = 620)	Normal‐weight (*n* = 5534)	Overweight (*n* = 3135)	Obesity (*n* = 1232)	*p*‐value
Gender, *n* (%)						< 0.001
Male	5035 (47.86)	298 (48.06)	2981 (53.87)	1332 (42.49)	424 (34.42)	
Female	5486 (52.14)	322 (51.94)	2553 (46.13)	1803 (57.51)	808 (65.58)	
Residence, *n* (%)						< 0.001
Rural	8640 (82.12)	553 (89.19)	4715 (85.2)	2424 (77.32)	948 (76.95)	
Urban	1881 (17.88)	67 (10.81)	819 (14.80)	711 (22.68)	284 (23.05)	
Education level, *n* (%)						< 0.001
Elementary school or below	7046 (66.97)	494 (79.68)	3830 (69.21)	1937 (61.79)	785 (63.72)	
Middle school	2290 (21.77)	85 (13.71)	1136 (20.53)	762 (24.31)	307 (24.92)	
High school	1022 (9.71)	40 (6.45)	489 (8.84)	371 (11.83)	122 (9.90)	
College or above	163 (1.55)	1 (0.16)	79 (1.43)	65 (2.07)	18 (1.46)	
Marital status, *n* (%)						< 0.001
Married	9446 (89.78)	525 (84.68)	4897 (88.49)	2886 (92.06)	1138 (92.37)	
Not married	1075 (10.22)	95 (15.32)	637 (11.51)	249 (7.94)	94 (7.63)	
Age (year), mean ± SD	57.66 ± 8.41	62.31 ± 8.97	58.03 ± 8.42	56.72 ± 8.08	56.06 ± 7.96	< 0.001
CRP (mg/L), median (IQR)	1.02 (0.54, 2.09)	0.82 (0.45, 1.80)	0.88 (0.49, 1.87)	1.12 (0.62, 2.12)	1.47 (0.81, 2.93)	< 0.001
Health insurance, *n* (%)						< 0.001
Urban employee medical insurance	1013 (9.63)	34 (5.48)	432 (7.81)	388 (12.38)	159 (12.91)	
Urban and rural resident medical insurance	8812 (83.76)	538 (86.77)	4756 (85.94)	2524 (80.51)	994 (80.68)	
Other medical insurance	105 (1.00)	2 (0.32)	50 (0.90)	39 (1.24)	14 (1.14)	
No insurance	591 (5.62)	46 (7.42)	296 (5.35)	184 (5.87)	65 (5.28)	
Household consumption (yuan), median (IQR)	16280.00 (8980.00, 27280.00)	12780.00 (7007.00, 23443.50)	15270.00 (8510.00, 25735.00)	17908.00 (10065.00, 29850.00)	17742.00 (9855.00, 30036.00)	< 0.001
Hypertension, *n* (%)						< 0.001
No	6497 (61.75)	455 (73.39)	3844 (69.46)	1732 (55.25)	466 (37.82)	
Yes	4024 (38.25)	165 (26.61)	1690 (30.54)	1403 (44.75)	766 (62.18)	
Dyslipidemia, *n* (%)						< 0.001
No	7838 (74.50)	541 (87.26)	4496 (81.24)	2093 (66.76)	708 (57.47)	
Yes	2683 (25.50)	79 (12.74)	1038 (18.76)	1042 (33.24)	524 (42.53)	
Diabetes, *n* (%)						< 0.001
No	9758 (92.75)	601 (96.94)	5271 (95.25)	2827 (90.18)	1059 (85.96)	
Yes	763 (7.25)	19 (3.06)	263 (4.75)	308 (9.82)	173 (14.04)	
Cancer or malignant tumor, *n* (%)						0.024
No	10,419 (99.03)	610 (98.39)	5492 (99.24)	3104 (99.01)	1213 (98.46)	
Yes	102 (0.97)	10 (1.61)	42 (0.76)	31 (0.99)	19 (1.54)	
Chronic lung diseases, *n* (%)						< 0.001
No	9499 (90.29)	508 (81.94)	4990 (90.17)	2878 (91.8)	1123 (91.15)	
Yes	1022 (9.71)	112 (18.06)	544 (9.83)	257 (8.20)	109 (8.85)	
Liver diseases, *n* (%)						0.651
No	10,101 (96.01)	599 (96.61)	5320 (96.13)	3000 (95.69)	1182 (95.94)	
Yes	420 (3.99)	21 (3.39)	214 (3.87)	135 (4.31)	50 (4.06)	
Heart diseases, *n* (%)						< 0.001
No	9352 (88.89)	558 (90)	5053 (91.31)	2726 (86.95)	1015 (82.39)	
Yes	1169 (11.11)	62 (10.00)	481 (8.69)	409 (13.05)	217 (17.61)	
Stroke, *n* (%)						< 0.001
No	10,338 (98.26)	611 (98.55)	5464 (98.74)	3070 (97.93)	1193 (96.83)	
Yes	183 (1.74)	9 (1.45)	70 (1.26)	65 (2.07)	39 (3.17)	
Kidney diseases, *n* (%)						0.044
No	9838 (93.51)	567 (91.45)	5161 (93.26)	2945 (93.94)	1165 (94.56)	
Yes	683 (6.49)	53 (8.55)	373 (6.74)	190 (6.06)	67 (5.44)	
Stomach or other digestive diseases, *n* (%)						< 0.001
No	8028 (76.30)	428 (69.03)	4190 (75.71)	2408 (76.81)	1002 (81.33)	
Yes	2493 (23.70)	192 (30.97)	1344 (24.29)	727 (23.19)	230 (18.67)	
Memory related diseases, *n* (%)						0.583
No	10,420 (99.04)	612 (98.71)	5487 (99.15)	3103 (98.98)	1218 (98.86)	
Yes	101 (0.96)	8 (1.29)	47 (0.85)	32 (1.02)	14 (1.14)	
Arthritis or rheumatism, *n* (%)						0.019
No	6841 (65.02)	390 (62.9)	3642 (65.81)	2052 (65.45)	757 (61.44)	
Yes	3680 (34.98)	230 (37.10)	1892 (34.19)	1083 (34.55)	475 (38.56)	
Asthma, *n* (%)						0.003
No	10,167 (96.64)	583 (94.03)	5359 (96.84)	3031 (96.68)	1194 (96.92)	
Yes	354 (3.36)	37 (5.97)	175 (3.16)	104 (3.32)	38 (3.08)	
Physical activities, *n* (%)						< 0.001
Vigorous activities	4066 (38.65)	264 (42.58)	2384 (43.08)	1086 (34.64)	332 (26.95)	
Moderate activities	3332 (31.67)	198 (31.94)	1668 (30.14)	1054 (33.62)	412 (33.44)	
Other activities	3123 (29.68)	158 (25.48)	1482 (26.78)	995 (31.74)	488 (39.61)	
Social activities, *n* (%)						< 0.001
No	5098 (48.46)	374 (60.32)	2794 (50.49)	1402 (44.72)	528 (42.86)	
Yes	5423 (51.54)	246 (39.68)	2740 (49.51)	1733 (55.28)	704 (57.14)	
Smoking status, *n* (%)						< 0.001
Current smokers	3293 (31.30)	241 (38.87)	2072 (37.44)	752 (23.99)	228 (18.51)	
Past smokers	867 (8.24)	44 (7.10)	420 (7.59)	293 (9.35)	110 (8.93)	
Never smokers	6361 (60.46)	335 (54.03)	3042 (54.97)	2090 (66.67)	894 (72.56)	
Drinking status, *n* (%)						< 0.001
Drink more than once a month	2710 (25.76)	153 (24.68)	1610 (29.09)	732 (23.35)	215 (17.45)	
Drink but less than once a month	849 (8.07)	41 (6.61)	478 (8.64)	240 (7.66)	90 (7.31)	
Do not drink	6962 (66.17)	426 (68.71)	3446 (62.27)	2163 (69.00)	927 (75.24)	
Self‐rated health, *n* (%)						< 0.001
Good	2465 (23.43)	104 (16.77)	1290 (23.31)	791 (25.23)	280 (22.73)	
Fair	5219 (49.61)	261 (42.10)	2797 (50.54)	1555 (49.60)	606 (49.19)	
Poor	2837 (26.97)	255 (41.13)	1447 (26.15)	789 (25.17)	346 (28.08)	

CRP, C‐reactive protein; BMI, body mass index; SD, standard deviation; IQR, interquartile range.

### 3.2 Trajectories of Depressive Symptoms

**FIGURE 2:**
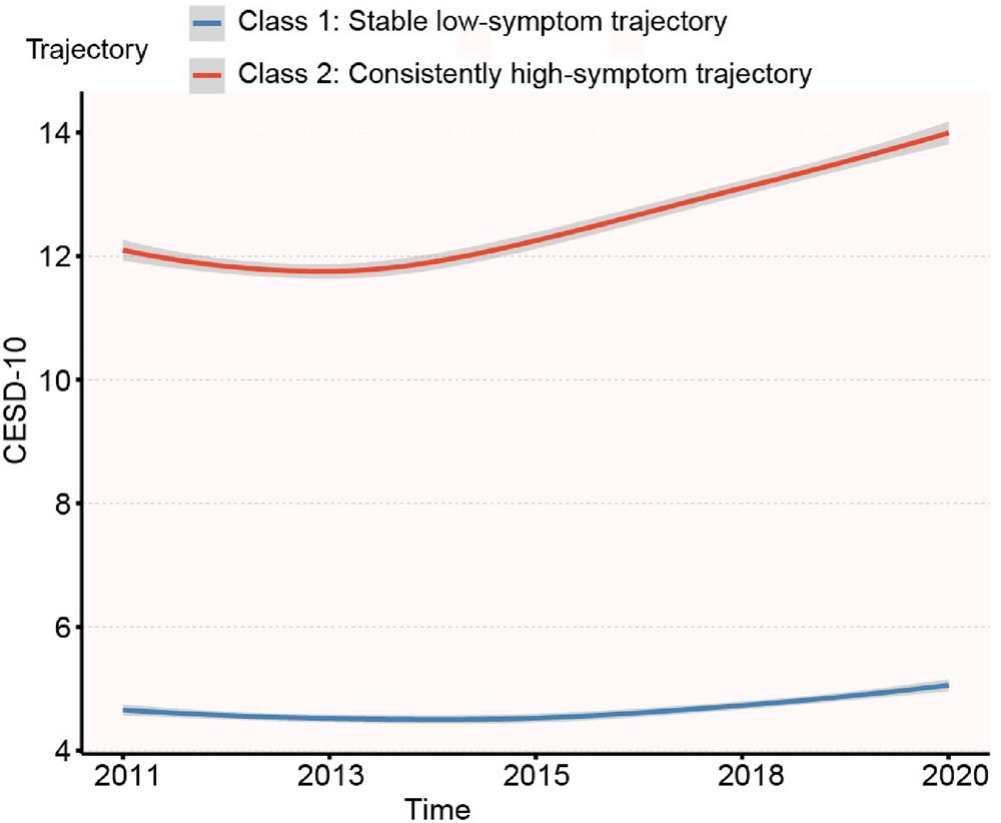
Trajectories of depressive symptoms.

A sequence of trajectory models varied from 2 to 6 classes were evaluated to determine the best‐fitting model characterizing the heterogeneity in dynamic change of depressive symptoms. While higher‐order trajectory models (3–6 classes) demonstrated progressive optimization of BIC and AIC, these models exhibited critical validity concerns: multiple trajectories contained class proportions <5% of the cohort and AvePP <0.70 (Table 2). Consequently, the parsimonious 2‐trajectory model was retained for subsequent analyses. Figure 2 illustrates two mutually exclusive trajectories identified in the study: one characterized by stable low‐symptom trajectory (*N* = 5467, 51.96%) and the other by consistently high‐symptom trajectory (*N* = 5054, 48.04%).

**TABLE 2:** Different depressive‐symptom trajectory models.

Group	AIC	BIC	Class proportion (%)						AvePP					
			Class 1	Class 2	Class 3	Class 4	Class 5	Class 6	Class 1	Class 2	Class 3	Class 4	Class 5	Class 6
2	279485.97	279594.89	51.96	48.04	NA	NA	NA	NA	0.85	0.91	NA	NA	NA	NA
3	278864.23	279009.45	40.07	25.18	34.75	NA	NA	NA	0.83	0.86	0.69	NA	NA	NA
4	278819.42	279000.95	38.27	10.05	20.42	31.27	NA	NA	0.77	0.45	0.83	0.61	NA	NA
5	278708.26	278926.09	39.54	13.15	14.25	5.08	27.98	NA	0.84	0.67	0.64	0.77	0.61	NA
6	278651.53	278905.67	38.68	20.48	9.09	13.57	11.14	7.04	0.81	0.52	0.52	0.77	0.61	0.55

NA, not available; AIC, Akaike Information Criterion; BIC, Bayesian Information Criterion; AvePP, average posterior probability.

The baseline demographic characteristics stratified by depressive symptom trajectory groups were summarized in Table S1. When compared to the stable low‐symptom trajectory group, participants in the consistently high‐symptom trajectory group exhibited significantly higher probabilities of: (a) demographic characteristics including female gender and unmarried status; (b) socioeconomic disadvantages such as lower educational attainment, reduced household consumption, and lack of health insurance coverage; (c) objective health conditions (chronic disease diagnoses); (d) subjective health perceptions (poor self‐rated health); and (e) behavioral factors including social activity disengagement.

### 3.3 Association Between BMI Categories and Depressive Symptom Trajectories

Table 3 presented the multivariable‐adjusted logistic regression analyses assessing the relationship between BMI categories and depressive symptom trajectory classifications. Relative to normal‐weight individuals, the adjusted ORs were 1.29 (95% CI: 1.07–1.56, *p* = 0.009) for underweight, 0.94 (95% CI: 0.85–1.04, *p* = 0.248) for overweight, and 0.89 (95% CI: 0.77–1.02, *p* = 0.100) for obesity. These associations remained robust in models with different covariate adjustments.

**TABLE 3:** Association between BMI categories and depressive symptom trajectories.

BMI	Event (%)	Model 1		Model 2		Model 3		Model 4	
		OR (95% CI)	*p*‐value	OR (95% CI)	*p*‐value	OR (95% CI)	*p*‐value	OR (95% CI)	*p*‐value
Normal‐weight	2669 (48.23)	1(Ref)		1 (Ref)		1 (Ref)		1 (Ref)	
Underweight	382 (61.61)	1.72 (1.45–2.04)	<0.001	1.55 (1.29–1.85)	<0.001	1.31 (1.09–1.58)	0.005	1.29 (1.07–1.56)	0.009
Overweight	1430 (45.61)	0.90 (0.82–0.98)	0.019	0.92 (0.83–1.00)	0.063	0.95 (0.86–1.05)	0.333	0.94 (0.85–1.04)	0.248
Obesity	573 (46.51)	0.93 (0.82–1.06)	0.275	0.89 (0.78–1.02)	0.088	0.90 (0.78–1.03)	0.138	0.89 (0.77–1.02)	0.100
Trend test			0.033		0.034		0.146		0.122

CI, confidence interval; OR, odds ratio.

*Note*: Model 1 was unadjusted; Model 2 was adjusted for sociodemographic characteristics; Model 3 was further adjusted for health‐related behaviors and factors; and Model 4 was additionally adjusted for chronic diseases. Estimates were from multivariable logistic regressions with the low‐symptom trajectory group as the reference. Coefficients represent ORs for each BMI category versus normal weight, for the low‐symptom group relative to the high‐symptom group.

### 3.4 Stratified and Sensitivity Analyses

**FIGURE 3:**
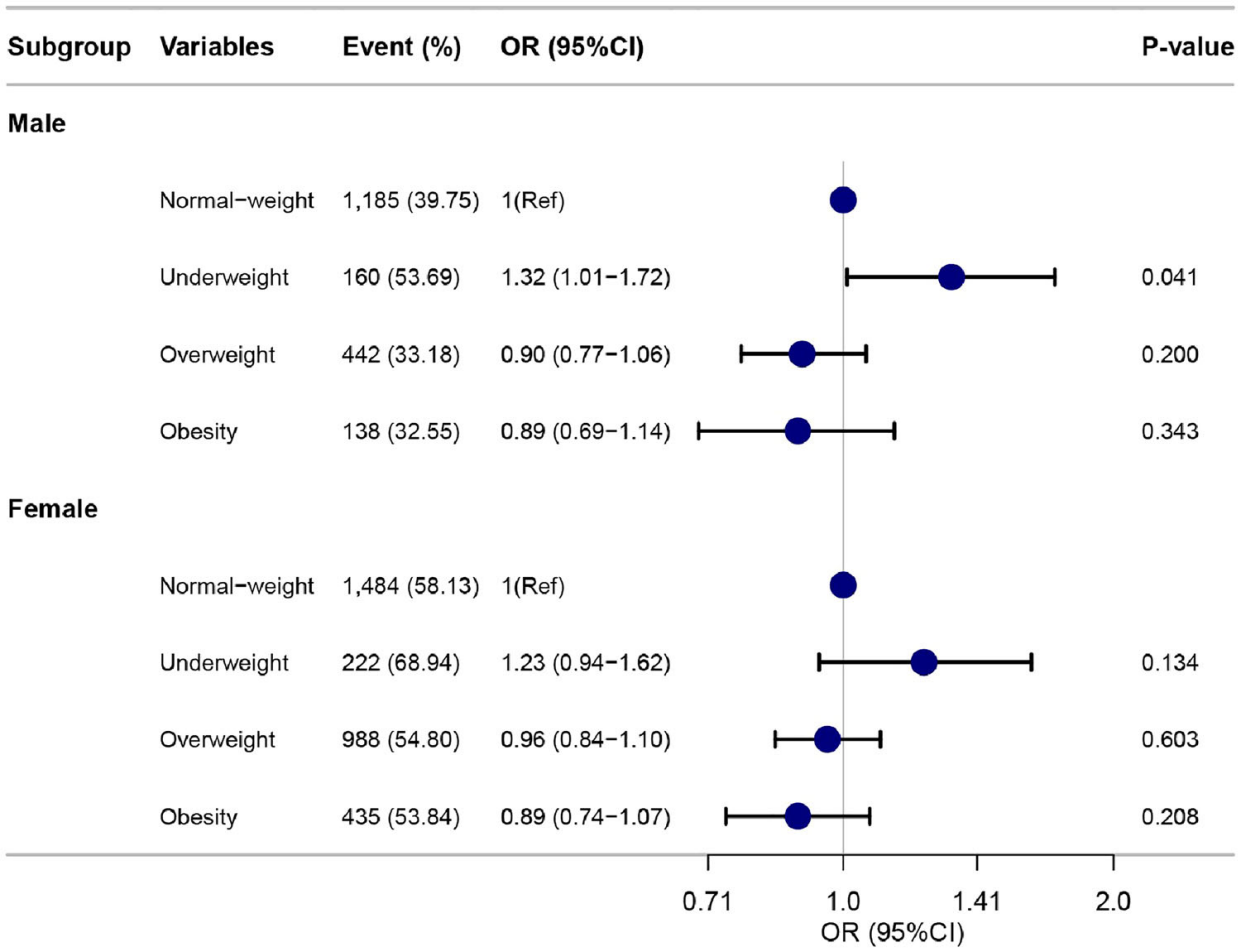
Stratified analysis by gender for the association between BMI categories and depressive symptom trajectories. Stratified model was adjusted for sociodemographic characteristics, health‐related behaviors and factors, and chronic diseases, with the exception of gender, which served as the stratification variable.

The association between BMI categories and longitudinal trajectories of depressive symptoms demonstrated significant gender‐specific variations (Figure 3). Among male participants, underweight status exhibited a positive association with the consistently high‐symptom trajectory group (adjusted OR = 1.32, 95% CI: 1.01–1.72, *p* = 0.041). In contrast, this association was not statistically significant in the female cohort (adjusted OR = 1.23, 95% CI: 0.94–1.62, *p* = 0.134). The results of sensitivity analyses were in line with our main findings, underscoring the robustness and stability of our results (Tables S2 and 3). After excluding individuals with missing covariate data, underweight status was positively associated with the consistently high‐symptom trajectory group (adjusted OR = 1.31, 95% CI: 1.05–1.65, *p* = 0.018). Likewise, in analyses that excluded participants with memory‐related disorders, underweight remained significantly associated with the high‐symptom trajectory (adjusted OR = 1.29, 95% CI: 1.07–1.56, *p* = 0.009). The *E*‐value for the association of underweight and consistently high‐symptom trajectory (adjusted OR = 1.29) was 1.53.

## 4 Discussion

This longitudinal study investigated depressive symptom trajectories across five waves of follow‐up in a nationally representative cohort of 10,521 middle‐aged and older adults in China. Additionally, we examined the association between baseline BMI and subsequent depressive symptom trajectories. The GBTM analysis revealed two statistically distinct depressive symptom trajectories: (1) a stable low‐symptom trajectory, and (2) a consistently high‐symptom trajectory. The multivariate analyses demonstrated that underweight status was associated with a greater odds of belonging to the consistently high‐symptom trajectory when compared to normal‐weight individuals. Notably, this association demonstrated apparent differences by gender, though the effect estimates for males and females were broadly similar, with overlapping confidence intervals. This sex‐specific differential persisted after adjusting for potential confounding variables including sociodemographic characteristics, health‐related behaviors and factors, and chronic diseases. Our findings carry substantial implications for both clinical practice and public health policy. In particular, the identification of underweight status as a determinant of persistent depressive symptoms among middle‐aged and older males highlights the necessity of embedding nutritional screening and targeted interventions within mental health care frameworks for this demographic.

Analysis of baseline characteristics revealed marked differences in sociodemographic and health‐related variables across both BMI categories and depressive symptom trajectory groups. These variables may function as confounders or effect modifiers in the association between BMI and depressive symptoms. For instance, although chronic diseases can independently increase depression risk, the significant association between underweight status and depressive symptoms persisted after multivariable adjustment, underscoring its independence from comorbid disease burden. While these baseline characteristics are not the primary focus of our analysis, their integration enriches the interpretive framework for our findings and emphasizes the need for tailored interventions that jointly target nutritional status and the broader social and clinical determinants of mental health in this population.

Our findings demonstrating elevated depressive symptom odds among underweight individuals align with existing epidemiological evidence. Notably, a recent large‐scale cross‐sectional study (*N* = 9995) conducted during the 2020–2021 period reported comparable results (Cui et al. 2024), with underweight participants exhibiting increased depressive symptom odds (adjusted OR = 1.35, 95% CI: 1.11–1.65) relative to normal‐weight participants (Cui et al. 2024). Furthermore, another cross‐sectional study using data from the 2017–2018 China Longitudinal Healthy Longevity Survey (CLHLS) showed that underweight was independently associated with depression symptoms (OR = 1.23, 95% CI: 1.05–1.45, *p* = 0.013) after adjusting potential confounding factors (Qiao et al. 2024). The gender‐stratified analysis yielded differential associations between BMI and depressive symptoms (Qiao et al. 2024). Among male participants, there was a positive association between underweight status and depressive symptoms (adjusted OR = 1.55, 95% CI: 1.19–2.03, *p* = 0.001) (Qiao et al. 2024). In contrast, this association was not statistically significant in the female subgroup (adjusted OR = 1.08, 95% CI: 0.88–1.33, *p* = 0.464) (Qiao et al. 2024). Additionally, a longitudinal analysis utilizing data from the China Family Panel Studies (CFPS) cohort revealed a significant association between BMI and depressive symptoms (Zhao et al. 2023). After controlling for potential confounders, both progression from normal weight to underweight (RR = 1.14, 95% CI: 1.07–1.23) and sustained underweight status (RR = 1.15, 95% CI: 1.08–1.24) were positively associated with depressive symptoms in middle‐aged participants (Zhao et al. 2023). However, some studies reported inconsistent findings (Luo et al. 2018; Mulugeta et al. 2018). In a longitudinal analysis using data from the British birth cohort, underweight was not associated with depressive symptoms in either gender (*p* >0.05) (Mulugeta A et al. 2018). However, obesity demonstrated a significant positive association with depressive symptoms specifically among female participants (OR = 1.38, 95% CI: 1.07–1.77) (Mulugeta et al. 2018). Moreover, according to a cohort study using data from the 2011–2015 CHARLS data, underweight was not associated with depressive symptoms in either male or female participants when compared to normal‐weight individuals (*p* > 0.05) (Luo et al. 2018). However, obesity exhibited a significant negative association with depressive symptoms specifically among male individuals (OR = 0.51, 95% CI: 0.35–0.74, *p* < 0.001) (Luo et al. 2018). The discrepancy between our findings and those of previous studies may be attributable to both psychosocial and sex‐specific biological factors. Specifically, men may perceive underweight status more negatively as an undesirable physical attribute than women, potentially owing to societal norms that associate masculinity with muscularity and physical robustness. Such body image dissatisfaction may precipitate social devaluation, internalized weight bias, and diminished self‐esteem, ultimately contributing to depressive symptomatology (Darimont et al. 2020; Jackson et al. 2016). In addition, sex hormone regulation may also underlie the differential associations observed between men and women (Jackson et al. 2016).

The observed inconsistencies across studies may be attributed to several methodological and demographic factors: population heterogeneity in demographic characteristics (including ethnic composition, geographic distribution, age demographics, and sex representation), different adjustment covariates and study designs, residual confounding from unmeasured covariates, and variability in depression symptom assessment methodologies. Beyond methodological differences across studies, the observed association between underweight and depressive trajectories may reflect complex biological and behavioral pathways (Djordjevic et al. 2022; Ke et al. 2025). Appetite dysregulation and weight change are hallmark features of depressive disorders, and accumulating evidence suggests that alterations in inflammatory markers, neuroendocrine function, and metabolic hormones could mediate this relationship (Darimont et al. 2020; Djordjevic et al. 2022; Ke et al. 2025). In addition, It is unclear whether depressive symptoms resulted from reduced food intake or if depressive symptoms led to decreased food intake, considering that depressive symptoms can decrease individuals’ appetite. Moreover, the stronger effect in males may indicate sex‐specific biological responses or differential reporting of somatic symptoms (Jackson et al. 2016). Our longitudinal study utilizes data from a nationally representative cohort to examine the association between BMI and temporal progression of depressive symptoms, rather than relying on a single measurement. This methodological approach enables more robust identification of potential associations between BMI and the development of depressive symptomatology. Notably, a bidirectional Mendelian randomization analysis revealed a statistically significant association between lower BMI and increased odds of depression among individuals of East Asian descent (OR = 1.14, 95% CI: 1.02–1.28, *p* = 0.021) (Chen et al. 2025). This genetic evidence corroborates our observational findings, thereby strengthening the validity of our conclusions regarding the relationship of BMI and depressive symptoms in this population. Furthermore, we performed gender‐stratified analyses, which are essential given the well‐documented sex differences in both depressive symptomatology and body weight perception. Our finding that underweight status was more strongly associated with depressive symptoms among males than among females adds novel evidence to the current literature and offers an empirical foundation for the design of sex‐specific clinical interventions.

It is important to acknowledge that the two identified depressive symptom trajectories differ primarily in symptom severity rather than in the temporal shape of symptom progression. Both the stable low‐symptom and consistently high‐symptom groups demonstrated relatively flat trajectories over the 9‐year follow‐up period, with no clear evidence of divergence or convergence in symptom levels over time. This observation aligns with previous longitudinal studies that have similarly identified severity‐based rather than shape‐based trajectory distinctions (Malhotra et al. 2016; Wang et al. 2023). Although our GBTM analysis successfully captured meaningful heterogeneity in the longitudinal course of depressive symptoms, the absence of distinct temporal patterns—such as increasing, decreasing, or curvilinear trajectories—suggests that depressive symptomatology in this population is characterized predominantly by stable individual differences in symptom burden. Nevertheless, the severity‐based nature of the trajectory distinction should be considered when interpreting our results, and future studies with longer follow‐up periods or different populations may be better positioned to detect trajectories with more pronounced temporal variability.

Mechanisms underlying the association between underweight and depressive symptoms remains unclear; however, molecular studies have provided some evidence for the involvement of leptin deficiency (Djordjevic et al. 2022; Ke et al. 2025). Emerging mechanistic evidence suggests that underweight status induces hypoleptinemia, which may mediate depressive pathophysiology through neurotrophic pathway dysregulation (Djordjevic et al. 2022). Experimental studies demonstrated that leptin administration dose‐dependently reverses depressive‐like behaviors in chronic restraint stress models, concomitant with significant upregulation of brain derived neurotrophic factor and tyrosine kinase receptor B (Ke et al. 2025). Moreover, the underweight condition, as a clinically significant anthropometric characteristic, may contribute to elevated depression risk through multiple psychosocial pathways, including impaired social functioning, negative body image perception, and diminished self‐esteem (Darimont et al. 2020; Jackson et al. 2016). Males demonstrated stronger negative perceptions of underweight status as an undesirable physical characteristic compared to their female counterparts (Darimont et al. 2020; Jackson et al. 2016). This gender‐specific body image evaluation pattern provides a plausible explanation for the observed differential association between underweight status and depressive symptoms.

Our study has several key strengths. First, we employed a nationally representative dataset characterized by substantial sample size and comprehensive racial/ethnic diversity, enhancing the representativeness and credibility of our findings. Second, our longitudinal analysis utilized the GBTM approach to systematically examine developmental patterns of depressive symptomatology across multiple time points. This technique provides a valuable longitudinal perspective that captures heterogeneity in symptom courses, allowing for more nuanced characterization of depressive symptom patterns than single time measurement. Third, to examine potential gender‐specific associations, we performed a stratified analysis evaluating the relationship between BMI and depressive symptoms across male and female subgroups. This study still has some limitations. First, depressive symptoms were assessed using the CESD‐10, a self‐report instrument that may introduce potential information bias due to its subjective nature. Nevertheless, the CESD‐10 has demonstrated robust psychometric properties, with established reliability (Cronbach's *α* = 0.78–0.79) and validated construct validity in multiple population‐based studies (Zheng et al. 2025; Jiang et al. 2025; Wang et al. 2024). Second, while the observed associations maintained statistical significance following adjustment for multiple relevant variables, the potential influence of residual confounding factors cannot be entirely excluded due to the observational nature of this study. However, the *E*‐value for the association of underweight and consistently high‐symptom trajectory (adjusted OR = 1.29) was 1.53, meaning that an unmeasured confounder would need to be associated with both underweight status and depressive symptoms by an OR of at least 1.53 (beyond the measured confounders) to fully explain away the observed association. This suggests that our findings are reasonably robust to unmeasured confounding. Third, in the CHARLS, memory‐related diseases—including Alzheimer's disease, brain atrophy, and Parkinson's disease—were identified based on physician‐diagnosed criteria. As these conditions may confound the estimates, we conducted a sensitivity analysis excluding such individuals and found that the results remained materially consistent with our main findings. Fourth, this cohort analysis allows for the identification of associations but does not establish a temporal or causal relationship between BMI and depressive symptoms. Finally, the present study included middle‐aged and elderly people aged 45 and above in China. Given this demographic focus, the generalizability of our findings to other age groups or cultural contexts may be limited and requires careful consideration in interpretation. Consequently, future research is necessary to corroborate our current findings.

## 5 Conclusion

The present longitudinal investigation identified a significant association between underweight status and an elevated odds of consistently high‐symptom trajectory in middle‐aged and older Chinese adults. Notably, this relationship exhibited gender‐specific variations, with underweight demonstrating a statistically significant positive correlation with depressive symptoms exclusively among male participants. Our findings provide empirical evidence supporting a longitudinal association between underweight status and depressive symptoms. The results suggest that interventions aimed at preventing or ameliorating underweight may contribute to reduced depressive symptomatology and enhanced healthy aging outcomes in middle‐aged and older adult populations.

## Author Contributions

**Dehua Zhao**: conceptualization, methodology, software, data curation, supervision, formal analysis, validation, investigation, visualization, project administration, Writing – review and editing, Writing – original draft. **Hongying Fan**: software, formal analysis. **Xiaoqing Long**: data curation, formal analysis, software, writing – review and editing.

## Funding

The authors have nothing to report.

## Ethics Statement

This study received ethical approval from the Biomedical Ethics Review Committee of Peking University (IRB00001052‐11015) and was conducted in accordance with the Declaration of Helsinki. Written informed consent was secured from all participants preceding data collection.

## Conflicts of Interest

The authors declare that they have no competing interests.

## Supporting information




**Supplementary Materials**: brb371744‐sup‐0001‐SuppMat.docx

## Data Availability

The data that support the findings of this study are available from the corresponding author upon reasonable request.
