# Supplementary material for: Association Between Body Mass Index and Depressive Symptom Trajectories Among Chinese Middle and Old Aged Adults: A Cohort Analysis Based on the CHARLS
Source: Brain Behav. 2026 Sep 15;16(9):e71744. doi: 10.1002/brb3.71744 (PMC13578923; doi:10.1002/brb3.71744)
Supplement: Supplementary file 1 [file BRB3-16-e71744-s001.docx]

**Supplementary TABLE 1** Baseline demographic characteristics stratified by depressive symptom trajectory groups

| Variables | Total  (n = 10,521) | Persistently low depressive symptoms (n = 5,467) | Consistently high depressive symptoms trajectory (n = 5,054) | *P*-value |
| --- | --- | --- | --- | --- |
| Gender, n (%) |  |  |  | < 0.001 |
| Male | 5,035 (47.86) | 3,110 (56.89) | 1,925 (38.09) |  |
| Female | 5,486 (52.14) | 2,357 (43.11) | 3,129 (61.91) |  |
| Residence, n (%) |  |  |  | < 0.001 |
| Rural | 8,640 (82.12) | 4,197 (76.77) | 4,443 (87.91) |  |
| Urban | 1,881 (17.88) | 1,270 (23.23) | 611 (12.09) |  |
| Education level, n (%) |  |  |  | < 0.001 |
| Elementary school or below | 7,046 (66.97) | 3,187 (58.30) | 3,859 (76.36) |  |
| Middle school | 2,290 (21.77) | 1,419 (25.96) | 871 (17.23) |  |
| High school | 1,022 ( 9.71) | 724 (13.24) | 298 (5.9) |  |
| College or above | 163 ( 1.55) | 137 (2.51) | 26 (0.51) |  |
| Marital status, n (%) |  |  |  | < 0.001 |
| Married | 9,446 (89.78) | 5,035 (92.10) | 4,411 (87.28) |  |
| Not married | 1,075 (10.22) | 432 (7.90) | 643 (12.72) |  |
| Health insurance, n (%) |  |  |  | < 0.001 |
| Urban employee medical insurance | 1,013 ( 9.63) | 757 (13.85) | 256 (5.07) |  |
| Urban and rural resident medical insurance | 8,812 (83.76) | 4,355 (79.66) | 4,457 (88.19) |  |
| Other medical insurance | 105 ( 1.00) | 73 (1.34) | 32 (0.63) |  |
| No insurance | 591 ( 5.62) | 282 (5.16) | 309 (6.11) |  |
| Hypertension, n (%) |  |  |  | 0.054 |
| No | 6,497 (61.75) | 3,424 (62.63) | 3,073 (60.80) |  |
| Yes | 4,024 (38.25) | 2,043 (37.37) | 1,981 (39.20) |  |
| Dyslipidemia, n (%) |  |  |  | 0.224 |
| No | 7,838 (74.50) | 4,100 (75.00) | 3,738 (73.96) |  |
| Yes | 2,683 (25.50) | 1,367 (25.00) | 1,316 (26.04) |  |
| Diabetes, n (%) |  |  |  | 0.001 |
| No | 9,758 (92.75) | 5,114 (93.54) | 4,644 (91.89) |  |
| Yes | 763 ( 7.25) | 353 (6.46) | 410 (8.11) |  |
| Cancer or malignant tumor, n (%) |  |  |  | 0.046 |
| No | 10,419 (99.03) | 5,424 (99.21) | 4,995 (98.83) |  |
| Yes | 102 ( 0.97) | 43 (0.79) | 59 (1.17) |  |
| Chronic lung diseases, n (%) |  |  |  | < 0.001 |
| No | 9,499 (90.29) | 5,073 (92.79) | 4,426 (87.57) |  |
| Yes | 1,022 ( 9.71) | 394 (7.21) | 628 (12.43) |  |
| Liver diseases, n (%) |  |  |  | < 0.001 |
| No | 10,101 (96.01) | 5,290 (96.76) | 4,811 (95.19) |  |
| Yes | 420 ( 3.99) | 177 (3.24) | 243 (4.81) |  |
| Heart diseases, n (%) |  |  |  | < 0.001 |
| No | 9,352 (88.89) | 4,981 (91.11) | 4,371 (86.49) |  |
| Yes | 1,169 (11.11) | 486 (8.89) | 683 (13.51) |  |
| Stroke, n (%) |  |  |  | < 0.001 |
| No | 10,338 (98.26) | 5,398 (98.74) | 4,940 (97.74) |  |
| Yes | 183 ( 1.74) | 69 (1.26) | 114 (2.26) |  |
| Kidney diseases, n (%) |  |  |  | < 0.001 |
| No | 9,838 (93.51) | 5,223 (95.54) | 4,615 (91.31) |  |
| Yes | 683 ( 6.49) | 244 (4.46) | 439 (8.69) |  |
| Stomach or other digestive diseases, n (%) |  |  |  | < 0.001 |
| No | 8,028 (76.30) | 4,492 (82.17) | 3,536 (69.96) |  |
| Yes | 2,493 (23.70) | 975 (17.83) | 1,518 (30.04) |  |
| Memory related diseases, n (%) |  |  |  | < 0.001 |
| No | 10,420 (99.04) | 5,433 (99.38) | 4,987 (98.67) |  |
| Yes | 101 ( 0.96) | 34 (0.62) | 67 (1.33) |  |
| Arthritis or rheumatism, n (%) |  |  |  | < 0.001 |
| No | 6,841 (65.02) | 4,039 (73.88) | 2,802 (55.44) |  |
| Yes | 3,680 (34.98) | 1,428 (26.12) | 2,252 (44.56) |  |
| Asthma, n (%) |  |  |  | < 0.001 |
| No | 10,167 (96.64) | 5,340 (97.68) | 4,827 (95.51) |  |
| Yes | 354 ( 3.36) | 127 (2.32) | 227 (4.49) |  |
| Physical activities, n (%) |  |  |  | 0.024 |
| Vigorous activities | 4,066 (38.65) | 2,046 (37.42) | 2,020 (39.97) |  |
| Moderate activities | 3,332 (31.67) | 1,777 (32.5) | 1,555 (30.77) |  |
| Other activities | 3,123 (29.68) | 1,644 (30.07) | 1,479 (29.26) |  |
| Social activities, n (%) |  |  |  | < 0.001 |
| No | 5,098 (48.46) | 2,423 (44.32) | 2,675 (52.93) |  |
| Yes | 5,423 (51.54) | 3,044 (55.68) | 2,379 (47.07) |  |
| Smoking status, n (%) |  |  |  | < 0.001 |
| Current smokers | 3,293 (31.30) | 1,894 (34.64) | 1,399 (27.68) |  |
| Past smokers | 867 ( 8.24) | 530 (9.69) | 337 (6.67) |  |
| Never smokers | 6,361 (60.46) | 3,043 (55.66) | 3,318 (65.65) |  |
| Drinking status, n (%) |  |  |  | < 0.001 |
| Drink more than once a month | 2,710 (25.76) | 1,676 (30.66) | 1,034 (20.46) |  |
| Drink but less than once a month | 849 ( 8.07) | 487 (8.91) | 362 (7.16) |  |
| Do not drink | 6,962 (66.17) | 3,304 (60.44) | 3,658 (72.38) |  |
| Self-rated health, n (%) |  |  |  | < 0.001 |
| Good | 2,465 (23.43) | 1,788 (32.71) | 677 (13.4) |  |
| Fair | 5,219 (49.61) | 2,905 (53.14) | 2,314 (45.79) |  |
| Poor | 2,837 (26.97) | 774 (14.16) | 2,063 (40.82) |  |
| CRP, Median (IQR) | 1.02 (0.54, 2.09) | 1.04 (0.55, 2.10) | 0.99 (0.54, 2.05) | 0.233 |
| Age (year), Mean ± SD | 57.66 ± 8.41 | 57.36 ± 8.55 | 57.98 ± 8.25 | < 0.001 |
| Household consumption (yuan), Median (IQR) | 16,280.00 (8,980.00, 27,280.00) | 17,800.00 (9,644.00, 29,740.00) | 14,716.00 (8,352.50, 24,970.00) | < 0.001 |

*Note:* CRP, C-reactive protein; BMI, body mass index; SD, standard deviation; IQR, interquartile range

**Supplementary TABLE 2** Association between BMI categories and depressive symptom trajectories after excluding individuals with missing covariate data

| BMI categories | Event (%) | OR (95%CI) | *P*-value |
| --- | --- | --- | --- |
| Normal-weight | 1,900 (49.13) | 1(Ref) |  |
| Underweight | 275 (62.50) | 1.31 (1.05-1.65) | 0.018 |
| Overweight | 1,031 (45.84) | 0.89 (0.79-1.00) | 0.060 |
| Obesity | 434 (48.12) | 0.90 (0.76-1.06) | 0.214 |
| Trend test |  |  | 0.087 |

*Note*: CI, confidence interval; OR, odds ratio. Multivariate regression model was adjusted for demographic and socioeconomic characteristics, health-related behaviors, health-related factors, and chronic diseases.

**Supplementary TABLE 3** Association between BMI categories and depressive symptom trajectories after excluding individuals with memory-related disorders

| BMI categories | Event (%) | OR (95%CI) | *P*-value |
| --- | --- | --- | --- |
| Normal-weight | 2,636 (48.04) | 1(Ref) |  |
| Underweight | 376 (61.44) | 1.29 (1.07-1.56) | 0.009 |
| Overweight | 1,411 (45.47) | 0.95 (0.86-1.05) | 0.299 |
| Obesity | 564 (46.31) | 0.89 (0.77-1.03) | 0.115 |
| Trend test |  |  | 0.151 |

*Note*: CI, confidence interval; OR, odds ratio. Multivariate regression model was adjusted for demographic and socioeconomic characteristics, health-related behaviors, health-related factors, and chronic diseases.
